# Supplementary material for: Prevalence of non-specific chronic low-back pain and risk factors among male soldiers in Saudi Arabia
Source: PeerJ. 2021 Oct 12;9:e12249. doi: 10.7717/peerj.12249 (PMC8519176; doi:10.7717/peerj.12249)
Supplement: Supplemental Information 2 [file peerj-09-12249-s002.docx]

Questions for eligibility

Would you please answer these questions for eligibility to participate in the study

1. Are you working in Hafer-Batin's military base? Yes/no?
2. Are you currently suffering from back pain? Yes/No
3. Is the duration of back pain 12 weeks or more? Yes/No
4. Do you provide your consent to review your medical records for any systemic disorder? Yes/No

If any of the answers are NO then the participant becomes ineligible to participate in the study.

Arabic translation of the questions below

هل يمكنك الإجابة على هذة الأسئلة من أجل أن تكون مؤهلاً للمشاركة في الدراسة .

١- هل تعمل في قاعدة حفر الباطن العسكرية ؟ ( نعم / لا )

٢- هل تُعاني حالياً من الآم الظهر ؟(نعم / لا )

٣- هل الآم الظهر مُستمرة ١٢ اسبوعاً أو أكثر ؟ ( نعم / لا )

٤ - هل تُقدم موافقتك على مراجعة سجلاتك الطبية بحثاً عن أي إظطراب جهازي ؟ ( نعم / لا)

إذا كانت أي من الإجابات ( لا ) يُصبح المشارك غير مؤهل للدراسة .
