# Supplementary material for: Prevalence of non-specific chronic low-back pain and risk factors among male soldiers in Saudi Arabia
Source: PeerJ. 2021 Oct 12;9:e12249. doi: 10.7717/peerj.12249 (PMC8519176; doi:10.7717/peerj.12249)
Supplement: Supplemental Information 3 [file peerj-09-12249-s003.pdf]

The Roland-Morris Disability Questionnaire

When your back hurts, you may find it difficult to do some of the things you normally do.

This list contains sentences that people have used to describe themselves when they have back pain. When you read them, you may find that some stand out because they describe you *today*.

As you read the list, think of yourself *today*. When you read a sentence that describes you today, put a tick against it. If the sentence does not describe you, then leave the space blank and go on to the next one. Remember, only tick the sentence if you are sure it describes you today.

1. I stay at home most of the time because of my back.

2. I change position frequently to try and get my back comfortable.

3. I walk more slowly than usual because of my back.

4. Because of my back I am not doing any of the jobs that I usually do around the house.

5. Because of my back, I use a handrail to get upstairs.

6. Because of my back, I lie down to rest more often.

7. Because of my back, I have to hold on to something to get out of an easy chair.

8. Because of my back, I try to get other people to do things for me.

9. I get dressed more slowly then usual because of my back.

10. I only stand for short periods of time because of my back.

11. Because of my back, I try not to bend or kneel down.

12. I find it difficult to get out of a chair because of my back.

13. My back is painful almost all the time.

14. I find it difficult to turn over in bed because of my back.

15. My appetite is not very good because of my back pain.

16. I have trouble putting on my socks (or stockings) because of the pain in my back.

17. I only walk short distances because of my back.

18. I sleep less well because of my back.

19. Because of my back pain, I get dressed with help from someone else.

20. I sit down for most of the day because of my back.

21. I avoid heavy jobs around the house because of my back.

22. Because of my back pain, I am more irritable and bad tempered with people than usual.

23. Because of my back, I go upstairs more slowly than usual.

24. I stay in bed most of the time because of my back.

Note to users:

This questionnaire is taken from: Roland MO, Morris RW. A study of the natural history of back pain. Part 1: Development of a reliable and sensitive measure of disability in low back pain. Spine 1983; 8: 141-144

The score of the RDQ is the total number of items checked – i.e. from a minimum of 0 to a maximum of 24.

It is acceptable to add boxes to indicate where patients should tick each item.

The questionnaire may be adapted for use on-line or by telephone.
