# Supplementary material for: Prevalence of non-specific chronic low-back pain and risk factors among male soldiers in Saudi Arabia
Source: PeerJ. 2021 Oct 12;9:e12249. doi: 10.7717/peerj.12249 (PMC8519176; doi:10.7717/peerj.12249)
Supplement: Supplemental Information 4 [file peerj-09-12249-s004.pdf]

مقياس درجة العجز الناتج عن ألم أسفل الظهر

إستبيان (رولاند موريس) لدرجة العجز

عندما تشعر بألم في الظهر، قد تجد صعوبة في القيام ببعض الأمور التي اعتدت علي القيام بها في الأحوال العادية.

تحتوي هذه القائمة علي بعض العبارات التي يستخدمها الناس في وصف حالتهم حينما يشعرون بألم الظهر. عندما تقرأ هذه العبارات، قد تلفت بعض العبارات انتباهك حيث تصف حالتك *اليوم*. عند قراءة هذه القائمة، فكر في حالتك *اليوم*. عندما تقرأ عبارة تصف حالتك *اليوم* ضع علامة "√" أمامها. إذا لم تصف العبارة حالتك، اترك المربع فارغا وانتقل إلي العبارة التالية.
تذكر، عليك وضع علامة فقط أمام العبارة إذا كنت متأكدا من أنها تصف حالتك *اليوم*.

1. أظل معظم اليوم في المنزل بسبب الألم في ظهري.
2. أقوم بتغيير وضعي كثيرا كي أشعر بالراحة في ظهري.
3. أمشي ببطء عن المعتاد بسبب الألم في ظهري.
4. بسبب الألم في ظهري، لا أقوم بأي من الأعمال التي اعتدت علي القيام بها في المنزل.
5. بسبب الألم في ظهري، أستعمل الدرابزين عند صعود السلالم.
6. بسبب الألم في ظهري، أرقد للحصول علي الراحة أكثر من المعتاد.
7. بسبب الألم في ظهري، أحتاج إلي الإمساك بشئ ما كي أتمكن من القيام من على كرسي منخفض.
8. بسبب الألم في ظهري، أحاول الاستعانة بالآخرين كي يقوموا بالأمور بدلا مني.
9. أرتدي ملابسي ببطء أكثر من المعتاد بسبب الألم في ظهري.
10. أقف لفترات قصيرة فقط بسبب الألم في ظهري.
11. بسبب الألم في ظهري، أحاول عدم الانحناء او الركوع.
12. أجد صعوبة في القيام من الكرسي بسبب الألم في ظهري.
13. ظهري يؤلمني معظم اليوم.
14. أجد صعوبة في التقلب في الفراش بسبب الألم في ظهري.
15. شهيتي ليست جيدة جدا للطعام بسبب الألم في ظهري.
16. أجد صعوبة في ارتداء الجوارب بسبب الألم في ظهري.
17. أمشي مسافات قصيرة فقط بسبب الألم في ظهري.
18. أنام أقل من المعتاد بسبب الألم في ظهري.
19. بسبب الألم في ظهري، أحتاج لمساعدة شخص آخرلارتداء ملابسي.
20. أجلس معظم أوقات اليوم بسبب الألم في ظهري.
21. أتجنب المهام الشاقة في المنزل بسبب الألم في ظهري.
22. بسبب الألم في ظهري، أصبحت عصبي ومعكر المزاج أكثرمن العادة مع الناس.
23. بسبب الألم في ظهري، أصعد السلالم بشكل أبطأ من المعتاد.
24. أظل في الفراش معظم اليوم بسبب الألم في ظهري.
